# Costs of treating multidrug-resistant TB in California in 2022

**DOI:** 10.5588/ijtld.23.0150

**Published:** 2023-11-01

**Authors:** S. Katrak, R. Wang, P. Barry

**Affiliations:** 1 Tuberculosis Control Branch, California Department of Public Health, Richmond, CA; 2 Division of Infectious Diseases, University of California, San Francisco, CA, USA

Dear Editor,

After years of treating multidrug-resistant TB (MDR-TB) with regimens that included five or more medications for 15–18 months, new clinical trial data supporting the use of 6-month, all-oral regimens have emerged.^1^,^2^ There are few cost estimates for treating MDR-TB in low-incidence settings,^3^–^5^ and those that exist either do not include new drug regimens or suggest that direct costs may be higher for the new regimens.^6^ We therefore performed an analysis to estimate the direct medical cost of treatment for MDR-TB using four different treatment regimens, including the new 6-month oral regimens, from the perspective of a public health clinic in California. The annual TB rate in California is 4.4/100,000,^7^ and of the roughly 2,000 people diagnosed with TB annually, 1–2% have MDR-TB. The California Department of Public Health (CDPH) provides clinical and public health consultation for patients with MDR-TB through the California MDR-TB Service.^8^

We used a micro-costing approach to estimate medication costs associated with a single episode of care for MDR-TB and combined these with published information on other direct medical costs. We estimated costs for a public health department treating an adult patient with MDR-TB with one of four regimens: an 18-month regimen, including injectable amikacin (first 6 months), oral levofloxacin, linezolid (LZD; 600 mg daily for 4 months, 300 mg daily for 14 months), cycloserine and ethionamide (‘INJ18’); an 18-month regimen, including bedaquiline (BDQ; first 6 months), levofloxacin, LZD (600 mg daily for 4 months, 300 mg daily for 14 months), cycloserine, and ethionamide (‘BDQ18’); a 6-month regimen of BDQ, pretomanid, and LZD (‘BPaL’); and a 6-month regimen comprising BDQ, pretomanid, LZD, and moxifloxacin (‘BPaLM’). Composition of the INJ18 and BDQ18 regimens were based on published guidance from the Centers for Disease Control and Prevention,^9^ assumed no susceptibility to first-line agents, and reflected local practice patterns, including difficulties in obtaining delamanid and clofazimine. For each MDR-TB treatment regimen, a standardized set of services and frequency of those services was itemized, including laboratory tests, imaging, clinic visits, clinical monitoring (e.g., vision tests, electrocardiogram), directly observed therapy (DOT), nurse case management, and for a subset of patients, inpatient hospitalization (Supplementary Table S1). Patients were assumed to have smear-positive pulmonary MDR-TB that converted to smear-negative after 2 weeks of treatment, and to culture-negative after 4 weeks of treatment, with retention in care and completion of assigned MDR-TB regimen. Additional assumptions included no treatment-related adverse events (AEs) necessitating regimen switch, and no relapses or treatment failures. Cost components were summed in 2022 U.S. dollars and rounded to the nearest US$1,000.

Inpatient cost estimates were based on a published cost estimate of MDR-TB therapy in the United States,^3^ adjusted to 2022 dollars. Inpatient costs were included for 46% of patients, reflecting California MDR-TB Service data showing that 46% of cases from 2018 to 2021 were hospitalized during initial diagnosis. Outpatient clinic costs were estimated using California Medicaid reimbursement schedule.^10^ Case management costs (e.g., nurse case manager, outreach worker time, or adherence incentives) were based on published estimates of 28 weeks of TB case management in a low-incidence U.S. state,^5^ corrected for 26 or 81 weeks of therapy and adjusted to 2022 dollars. Medication costs were estimated based on two types of drug pricing: mean 340B pricing available to three local California public health TB programs in 2022, and commercial wholesale acquisition cost (WAP) published in the 2022 Red Book database.^11^ Costs of laboratory tests and monitoring procedures were estimated based on California Medicaid reimbursement schedule;^10^ this category included bacteriology, molecular tests for drug resistance, HIV testing, chest X-ray, comprehensive metabolic panels, magnesium assays, thyroid stimulating hormone testing (for those on ethionamide), drug concentration assays, DOT, vision screening, electrograms (ECGs) (for BDQ-containing regimens); and for the INJ18 group, peripherally inserted central catheter line insertions, injections, audiograms, and vestibular examination. The analysis did not require human subjects review, according to CDPH policy and the U.S. Code of Federal Regulations, 45 CFR 46.101.

Costs associated with each regimen are presented in the Table. Total direct cost for MDR-TB care was US$86,000–94,000 for BPaL, US$86,000–96,000 for BPaLM, US$117,000–187,000 for INJ18, and US$127,000–203,000 for BDQ18. Hospitalization costs comprised the largest proportion of direct costs for BPaL/BPaLM, regardless of type of drug pricing (47–53%) but were also a substantial component of other regimens (22–39% for BDQ18 and INJ18). The cost of medications comprised roughly one third of the total cost of the BPaL/BPaLM regimens for both 340b and WAP pricing. BDQ was the largest contributor to cost of medications for BPaL and BPaLM, and accounted for 87–89% of drug costs, even with public health pricing. Outpatient monitoring, including DOT, laboratory testing, and imaging, was highest for BDQ18 and INJ18. Overall, BPaL and BPaLM were the least costly regimens for treating MDR-TB because of shorter duration and reduced costs of the drugs, but also case management, DOT and clinical monitoring. However, BDQ is expensive – even when 340b pricing was used, costs for BDQ comprised more than a quarter of all direct costs for 6-month regimens.

**Table:** Costs associated with an episode of MDR-TB care in a U.S. public health clinic, 2022*

	All-oral BDQ regimen, 18 months (‘BDQ18’)		Injectable regimen, 18 months (‘INJ18’)		BDQ, Pa, LZD, 6 months (‘BPaL’)		BDQ, Pa, LZD, MFX, 6 months (‘BPaLM’)	
	340b US$	WAP US$	340b US$	WAP US$	340b US$	WAP US$	340b US$	WAP US$
Initial hospital visit and care, *n* (%)	45,208 (36)	45,208 (22)	45,208 (39)	45,208 (24)	45,208 (53)	45,208 (48)	45,208 (53)	45,208 (47)
Outpatient care, *n* (%)	34,303 (27)	34,303 (17)	34,303 (29)	34,303 (18)	11,110 (13)	11,110 (12)	11,110 (13)	11,110 (12)
Case management for duration of treatment	33,820	33,820	33,820	33,820	10,856	33,820	10,856	33,820
Outpatient 60-min new patient visit	83	83	83	83	83	83	83	83
Outpatient 30-min established patient visits	400	400	400	400	172	172	172	172
Medications, total, *n* (%)	38,215 (30)	113,889 (56)	16,036 (14)	85,287 (46)	25,676 (30)	34,327 (36)	25,747 (30)	36,006 (37)
BDQ cost	22,839	30,000	—	—	22,839	30,000	22,839	30,000
Pa cost	—	—	—	—	2,738	3,463	2,738	3,463
All other drugs, cost	15,376	83,889	16,036	85,287	99	864	170	2,543
Laboratory tests, imaging, and monitoring procedures, *n* (%)	9,508 (7)	9,508 (5)	21,783 (19)	21,783 (12)	3,560 (4)	3,560 (4)	3,560 (4)	3,560 (4)
Episode of care, total	$127,000	$203,000	$117,000	$187,000	$86,000	$94,000	$86,000	$96,000

* Costs in 2022 U.S. dollars.

MDR-TB = multidrug-resistant TB; BDQ = bedaquiline; Pa = pretomanid; LZD = linezolid; MFX = moxifloxacin; 340b = 340b drug pricing; WAP = wholesale acquisition cost.

This analysis was limited to estimating direct costs of treatment using a healthcare perspective and was designed to avoid overestimating costs associated with an episode of MDR-TB care. By summing disease-specific medical expenditures and relying solely on the California Medicaid reimbursement schedule, we likely underestimated direct costs in terms of provider time, staff time, facility time, spillover costs associated with mental health and subspecialty care, as well as the costs of commonly encountered challenges such as drug side effects and treatment switches. Based on clinical trial data showing that 25–57% of patients on 6-month regimens experienced AEs Grade ≥3, the scenario presented for this analysis is clearly idealized; notably, rates of AE for 6-month regimens were consistently lower than those in traditional regimens, suggesting that AE-associated costs may be less for 6-month regimens.^1^,^2^

Even under ideal conditions of no medication AEs, no treatment failure and public sector pricing, MDR-TB continues to be expensive. However, our analysis suggests that treatment costs for 6-month oral regimens compare favorably to costs of treating hepatitis C with direct-acting antivirals.^12^ Our analysis is based in California; however, the cost of BDQ (a primary driver of the cost of the 6-month regimen) is the same across the United States because it is available through a single drug distributor. Although the BPaL regimen is likely cost-effective in higher incidence settings,^13^ analogous work has yet to be performed for the United States. Future work should focus on societal costs averted through treatment, as has been demonstrated for drug-susceptible TB.^14^ Our analysis suggests that despite the high cost of medications, the short BPaL/BPaLM regimens provide cost savings in a U.S. public health setting. Further cost savings would require a lower cost for BDQ or reduction in hospitalization.

## References

[1] Nyang’wa B-T (2022). A 24-week, all-oral regimen for rifampin-resistant tuberculosis. *N Engl J Med*.

[2] Conradie F (2020). Treatment of highly drug-resistant pulmonary tuberculosis. *N Engl J Med*.

[3] Marks S (2014). Treatment practices, outcomes, and costs of multidrug-resistant and extensively drug-resistant tuberculosis, United States, 2005–2007. *Emerg Infect Dis J*.

[4] Rajbhandary SS, Marks SM, Bock NN. (2004). Costs of patients hospitalized for multidrug-resistant tuberculosis. *Int J Tuberc Lung Dis*.

[5] Rubado DJ (2008). Determining the cost of tuberculosis case management in a low-incidence state. *Int J Tuberc Lung Dis*.

[6] Diel R (2021). Cost of multidrug resistant tuberculosis in Germany: An update. *Int J Infect Dis*.

[7] California Department of Public Health (2023). TB disease data and publications. https://www.cdph.ca.gov/Programs/CID/DCDC/Pages/TB-Disease-Data.aspx.

[8] Shah NS (2018). The California Multidrug-Resistant Tuberculosis Consult Service: a partnership of state and local programs. *Public Health Action*.

[9] Nahid P (2019). Treatment of drug-resistant tuberculosis. An Official ATS/CDC/ERS/IDSA Clinical Practice Guideline. *Am J Respir Crit Care Med*.

[10] Medi-Cal (2023). *Medi-Cal rates range display*.

[11] Merative Micromedex (2022). Red Book Online. https://www.micromedexsolutions.com.

[12] Kaplan DE (2022). Cost-effectiveness of direct-acting antivirals for chronic hepatitis C virus in the United States from a payer perspective. *J Manag Care Spec Pharm*.

[13] Gomez GB (2021). Cost-effectiveness of bedaquiline, pretomanid and linezolid for treatment of extensively drug-resistant tuberculosis in South Africa, Georgia and the Philippines. *BMJ Open*.

[14] Castro KG (2016). Estimating tuberculosis cases and their economic costs averted in the United States over the past two decades. *Int J Tuberc Lung Dis*.

