# Supplementary material for: Costs of treating multidrug-resistant TB in California in 2022
Source: Int J Tuberc Lung Dis. 2023 Nov 1;27(11):864–6. doi: 10.5588/ijtld.23.0150 (PMC10599414; doi:10.5588/ijtld.23.0150)
Supplement: Supplementary file 1 [file iutld_ijtld_23.0150_supplementarydata1.pdf]

http://dx.doi.org/10.5588/ijtld.23.0150

SUPPLEMENTARY DATA

Costs of treating multidrug-resistant TB in California in 2022

Supplementary Table S1. Unit costs and occurrences of items associated with an episode of MDR TB Care in a U.S. Public Health Clinic, 2022

Description	CPT code or Reference	Cost, 2022 U.S. dollars	Occurrences, All-oral regimen, 18 months (BDQ18)	Occurrences, Injectable regimen, 18 months (INJ18)	Occurrences, BDQ, Pa, LZD, 6 months (BPaL)	Occurrences, BDQ, Pa, LZD, MFX, 6 months (BPaLM)
**Initial Hospital Visit and Care**	Marks SM, 2014.[1]	$98,278	0.46	0.46	0.46	0.46
**Office/outpatient visit, new 60-87 min**	99205	$82.70	1	1	1	1
**Office/outpatient visit, established 30 min**	99215	$57.20	7	7	3	3
**Case management per week**	Rubado DJ, 2008.[2]	$417.53	26	26	81	81
**Bedaquiline, 26-week course**	340b pricing	$22,839.30	1	0	1	1
	WAP pricing	$30,000.00				
**Linezolid, 600 mg, 30-day supply**	340b pricing	$15.96	4	4	6	6
	WAP pricing	$143.39				
**Linezolid, 300 mg, 30-day supply**	340b pricing	$7.98	14	14	0	0

	WAP pricing	$71.70				
**Cycloserine, 250 mg bid, 30-day supply**	340b pricing	$268.65	18	18	0	0
	WAP pricing	$3,895.76				
**Levofloxacin 750 mg daily, 30-day supply**	340b pricing	$3.76	18	18	0	0
	WAP pricing	$116.07				
**Moxifloxacin 400 mg daily, 30-day supply**	340b pricing	$11.85	0	0	0	6
	WAP pricing	$279.83				
**Ethionamide 750 mg daily, 30-day supply**	340b pricing	$188.10	18	18	0	0
	WAP pricing	$560.51				
**Amikacin 900 mg IV 5x/week, 30-day supply**	340b pricing	$110.07	0	6	0	0
	WAP pricing	$233.00				
**Pretomanid 200 po daily, 30-day supply**	340b pricing	$456.31	0	0	6	6
	WAP pricing	$577.24				
**B6 50 md daily, 30-day supply**	340b pricing	$0.53	18	18	6	6
	WAP pricing	$0.53				

**Xray exam chest 1 view**	71045	$17.67	6	6	3	3
**Smear/Acid stain**	87206	$4.79	26	26	14	14
**Mycobacteria culture**	87116	$9.12	26	26	14	14
**DNA/RNA direct probe**	87149	$16.24	3	3	3	3
**Microbe susceptibility mycobacteria**	87190	$4.56	13	13	13	13
**MTB DNA Amp Probe (Xpert)**	87556	$33.34	1	1	1	1
**MTB DNA Quant (PSQ, MDDR)**	87557	$38.08	2	2	2	2
**Complete blood count**	85027	$5.71	21	21	9	9
**Comprehensive metabolic panel**	80053	$9.28	19	19	7	7
**Serum magnesium**	83735	$5.96	19	7	7	7
**Thyroid stimulating hormone**	84443	$14.76	7	7	0	0
**HIV**	87389	$20.26	1	1	1	1
**Drug level, cycloserine**	80299	$12.59	1	1	0	0
**Drug level, linezolid**	80299	$12.59	1	1	1	1
**Drug level, amikacin**	80150	$13.40	0	1	0	0
**DOT visits**	H0033	$19.23	391	391	130	130

**Insert PICC catheter**	36568	$68.56	0	1	0	0
**Maintain PICC catheter**	36573	$362.99	0	28	0	0
**Amikacin sulfate injection**	J0278	$5.52	0	108	0	0
**Visual acuity screening**	99173	$4.03	19	19	7	7
**Color vision examination**	92283	$16.79	19	19	7	7
**Audiology evaluation**	92557	$181.49	0	7	0	0
**Depression assessment (PHQ9)**	96127	$4.81	19	19	0	0
**Vestibular exam**	92540	$82.13	0	6	0	0
**Electrocardiogram complete**	93000	$28.70	9	0	9	9

[1] Marks SM, Flood J, Seaworth B, et al. Treatment practices, outcomes, and costs of multidrug-resistant and extensively drug-resistant tuberculosis, United States, 2005-2007. *Emerg Infect Dis*. 2014;20(5):812-821. doi:10.3201/eid2005.131037

[2] Rubado DJ, Choi D, Becker T, Winthrop K, Schafer S. Determining the cost of tuberculosis case management in a low-incidence state. Int J Tuberc Lung Dis. 2008 Mar;12(3):301-7. PMID: 18284836.

Abbreviations: 340b = 340b drug pricing, BDQ = bedaquiline, CPT = Current Procedural Terminology, DOT = directly observed therapy, LZD = linezolid, MDDR = molecular detection of drug resistance, MDR = multidrug-resistant, MFX = moxifloxacin, MTB = Mycobacterium tuberculosis, Pa = pretomanid, PHQ-9 = Patient health questionnaire-9, PICC = peripherally inserted central catheter, PSQ = pyrosequncing, TB = tuberculosis, Xprt = Gene Xprt PCR test, U.S. = United States, WAP = wholesale acquisition cost
